# Gas diffusion electrodes improve hydrogen gas mass transfer for a hydrogen oxidizing bioanode

**DOI:** 10.1002/jctb.5412

**Published:** 2017-10-03

**Authors:** Pau Rodenas, Fangqi Zhu, Annemiek ter Heijne, Tom Sleutels, Michel Saakes, Cees Buisman

**Affiliations:** ^1^ Wetsus, European centre of excellence for Sustainable Water Technology Leeuwarden The Netherlands; ^2^ Sub‐Department of Environmental Technology Wageningen University Wageningen The Netherlands

**Keywords:** BES, copper, gas diffusion electrode, MET

## Abstract

**Background:**

Bioelectrochemical systems (BESs) are capable of recovery of metals at a cathode through oxidation of organic substrate at an anode. Recently, also hydrogen gas was used as an electron donor for recovery of copper in BESs. Oxidation of hydrogen gas produced a current density of 0.8 A m^‐2^ and combined with Cu^2+^ reduction at the cathode, produced 0.25 W m^‐2^. The main factor limiting current production was the mass transfer of hydrogen to the biofilm due to the low solubility of hydrogen in the anolyte. Here, the mass transfer of hydrogen gas to the bioanode was improved by use of a gas diffusion electrode (GDE).

**Results:**

With the GDE, hydrogen was oxidized to produce a current density of 2.9 A m^‐2^ at an anode potential of –0.2 V. Addition of bicarbonate to the influent led to production of acetate, in addition to current. At a bicarbonate concentration of 50 mmol L^‐1^, current density increased to 10.7 A m^‐2^ at an anode potential of –0.2 V. This increase in current density could be due to oxidation of formed acetate in addition to oxidation of hydrogen, or enhanced growth of hydrogen oxidizing bacteria due to the availability of acetate as carbon source. The effect of mass transfer was further assessed through enhanced mixing and in combination with the addition of bicarbonate (50 mmol L^‐1^) current density increased further to 17.1 A m^‐2^.

**Conclusion:**

Hydrogen gas may offer opportunities as electron donor for bioanodes, with acetate as potential intermediate, at locations where excess hydrogen and no organics are available. © 2017 The Authors. *Journal of Chemical Technology & Biotechnology* published by John Wiley & Sons Ltd on behalf of Society of Chemical Industry.

## INTRODUCTION

For the electrochemical removal or recovery of heavy metals, e.g. copper, from wastewater, an electron donor is required. A suitable electron donor is water, which is oxidized to yield oxygen, protons, and electrons. The reduction of copper in combination with water oxidation, however, requires a theoretical minimum energy input of 0.10 kWh kg^‐1^ Cu. In practice, industrial copper plating requires 2.7 kWh kg^‐1^ Cu when water is used as electron donor due to a high overpotential for this reaction.^1^, ^2^ Previously it has been shown that bioelectrochemical systems (BESs) can be used for the recovery of metals, using electron donors present in wastewater such as acetate.^3^ The oxidation of organics such as acetate, gives an energetic advantage over water oxidation and even results in power production combined with copper recovery. For example, Rodenas *et al*. achieved a power production of 5.5 W m^‐2^ (0.081 kWh kg^‐1^ Cu) by using acetate as an electron donor at a current densitiy of 23 A m^‐2^.^4^ These current densities are low, however, when compared with the electroplating industry (100 A m^‐2^).^5^ Besides the lower current densities, another disadvantage of using wastewater as electron donor is that organics are not commonly available in hydrometallurgical or mining industries. On the other hand, these industries often produce hydrogen gas, that is considered a waste stream. For example, electroplating of metals such as zinc, cobalt, lead or nickel produces hydrogen while some other metallurgical industries use steam reforming of hydrocarbons to power the ovens or engines which also produces hydrogen as byproduct.^6^, ^7^, ^8^ Previous research showed that this hydrogen gas can be used as an alternative to acetate as an electron donor at the bioanode in BESs.^9^ Hydrogen gas was oxidized directly by bacteria on a graphite anode, eliminating the need for an expensive catalyst like platinum, which would be needed for electrochemical oxidation of hydrogen gas.^10^ When coupled to copper reduction, electricity was produced, resulting in power production of 0.25 W m^‐2^ (0.11 kWh kg^‐1^ Cu).^9^ The energy losses at the anode were partly explained by the limited solubility of hydrogen gas in water, causing mass transfer limitations. Enhancing mass transfer would result in higher current densities and higher energy recovery.^11^, ^12^ The main advantage of gas diffussion electrodes (GDEs) is that the fuel for the electrode reactions (hydrogen or oxygen gas) does not need to be dissolved into the electrolyte and can, therefore, be transferred faster to the electrode. The gas molecules react directly at, and release electrons to the electrode surface while the ions produced are released into the electrolyte. The electrolyte is required not only to have a watery environment for the microorganisms, but also provides micronutrients and allows ions to be transported to the membrane. Application of GDEs in combination with microorganisms is already well established for oxygen reducing cathodes.^13^

Overall, the aim of this study was to improve mass transfer of hydrogen gas to a bioanode by the use of a gas diffusion electrode to increase the current density produced from this hydrogen gas. To test its suitability, we analyzed the performance of a GDE for use as a hydrogen‐fed bioanode using polarization tests. The effect of enhanced mass transfer through mixing was assessed and the mechanisms of electricity production through addition of bicarbonate investigated.

## MATERIALS AND METHODS

### Experimental set up

Two plexiglass plates, each with a single flow channel, contained anolyte and catholyte and were separated by a Ralex anion exchange membrane with a surface area of 22 cm^2^ (MEGA a.s.,Stráž pod Ralskem, Czech Republic). This membrane surface area was used to normalise current production, which is equal to the projected anode surface area, since this allows for comparison with other studies.

**Figure 1:**
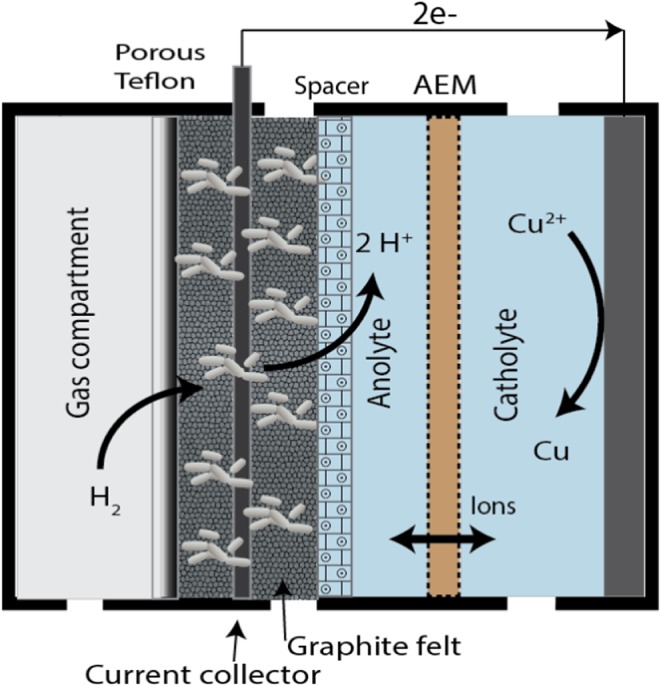
Schematic representation of the experimental set‐up with the gas diffusion electrode. The system contains from left to right the gas compartment where the hydrogen gas is fed to the system, hydrofobic layer (porous teflon) which allows the hydrogen gas to diffuse directly to the biofilm, electrode with biofilm, Pt current collector, spacer material, anolyte which contains the nutrients and is continuously fed to the system, membrane which keeps the anolyte and catholyte separated and allows ions to be transported, catholyte and cathode are depicted.

The cell (biotic and abiotic control) was constructed, as described by ter Heijne *et al*.^14^ One additional plexiglas plate with flow channel served as gas compartment and was placed adjacent to the anode (Fig. 1). This gas compartment was closed on the opposite side of the gas inlet to force the gas through the GDE into the anode compartment. The GDE consisted of a porous Teflon layer (POREX® Porous PTFE Porex Technologies GmbH, Germany) and two layers of graphite felt (anode) of 2 mm thickness on each side of a 510 μm Pt coated Ti‐mesh current collector (Magneto Special Anodes, B.V., Schiedam, The Netherlands). This GDE was separated from the anion exchange membrane by a spacer of 560 μm (PETEX, Sefar BV, Goor, Netherlands) and a 3 mm rubber between the GDE and membrane to allow a flow of anolyte.

The cathode material was graphite foil (1.0 g/cm^3^ density, 99% purity; Coidan Graphite Products Ltd, York, UK), which was pressed on the cathode current collector made of stainless steel.

Temperature and pH were continuously recorded (Endress + Hauser, Liquiline data logger) through pH electrodes (Endress + Hauser, CPS41 D) that were placed in the recirculation of anolyte and catholyte. Potentials were measured and reported vs Ag/AgCl reference electrodes, placed in anolyte and catholyte (+0.204 V vs NHE; QR400X QiS ProSense B.V. The Netherlands). Cell voltage was recorded simultaneously.

### Electron donor and electrolyte composition

The anolyte chamber was operated in a continuous mode and the catholyte in a batch mode. The anolyte was recirculated at 100 mL min^‐1^ via a recirculation bottle of 0.5 L. The catholyte was recirculated at the same rate via a 1 L bottle. The anode influent (1.5 mL min^‐1^), buffered at pH 7, consisted of. 0.68 g L^‐1^ KH_2_PO_4_, 0.87 g L^‐1^ K_2_HPO_4_, 0.74 g L^‐1^ KCl, 0.58 g L^‐1^ NaCl, 0.28 g L^‐1^ NH_4_Cl, 0.1 g L^‐1^ MgSO_4_.7H_2_O, 0.1 g L^‐1^ CaCl_2_·2H_2_O and 0.1 mL L^‐1^ of a trace element mixture.^15^ In all experiments, if not stated otherwise, the influent also contained 1 mmol L^‐1^ of NaHCO_3_.

The catholyte consisted of a 1 g L^‐1^ Cu^2+^ solution (prepared from CuCl_2_ and deionized water; pH 3). The cathode was kept anaerobic by flushing with nitrogen gas to avoid oxygen reduction interfering with the copper plating.

The cell was inoculated with 10 mL of effluent from a running microbial fuel cell fed with acetate containing a mixed culture enriched with Geobacter sp in previous experiments.^16^

### Experimental strategy

The experimental strategy consisted of four steps: (1) start‐up and characterization of the cell with acetate as electron donor; (2) hydrogen gas as an electron donor at two different inflow rates; (3) addition of bicarbonate to the anode influent at a constant hydrogen inflow rate; and (4) assesment of mass transfer through changes in circulation speed of the anolyte.

During start‐up the bioanode was fed with 10 mmol L^‐1^ acetate. After current density had stabilized (23 days), the current density was characterized at different anode potentials. The anode potential was changed through a programmed sequence from –0.45 to –0.20 V vs Ag/AgCl, in steps of 50 mV, each step lasting 120 min. This anode potential was controlled using a potentiostat (AUTOLAB 302 N, Metrohm Autolab B.V.,Utrecht, The Netherlands). In the second phase, hydrogen gas was used as an electron donor instead of acetate. First, the cell was fed with 3 mL min^‐1^ H_2_ inflow using a mass flow controller (Bronkhorst HIGH‐TECH BV, Ruurlo, The Netherlands), which was later increased to 10 mL min^‐1^. After that, the effect of bicarbonate in the influent on current production was investigated. The bicarbonate concentration was changed from 0 to 50 mmol L^‐1^. Finally, the effect of anolyte recirculation rate was studied. For the anolyte recirculation experments, the recirculation rate was changed from 50 mL min^‐1^ to 100, 150, and 200 mL min^‐1^.

### Analytical procedures

Bicarbonate concentration in the cells was determined using a total carbon analyzer (Shimadzu TOC‐VCPH) and the acetate concentration was measured using ion chromatography (Metrohm 761 Compact IC equipped with a conductivity detector and a Metrosep Organic Acids 6.1005.200 ion exclusion column). The amount of recoverd copper was not analysed, since the experiments were conducted under anode potential controlled conditions and therefore the current produced is only representative for the performance of the anode.

### Microbial community analysis

Amplification and high‐throughput sequencing were used to study the microbial community in the anolyte and on the anode. Environmental samples were sent to and analysed by GATC Biotech AG (Konstanz, Germany). The microbial community was established by amplicon sequencing of a 570 bp (excluding primer length) fragment of the V3‐V5 hypervariable region.^17^ PCR amplification was performed by using the 357F (CCTACGGGAGGCAGCAG) and 926R primer (CCGTCAATTCMTTTRAGT). Unique clusters are subjected to BLASTn^18^ analysis using non‐redundant 16S rRNA reference sequences with an E‐value cutoff of 1 × 10^‐6^. Reference 16S rRNA sequences are obtained from Ribosomal Database Project^19^ (RDP release 11, updated 11 September 2016). Only good quality and unique 16S rRNA sequences which have a taxonomic assignment are considered and used as a reference database to assign operational taxonomic unit (OTU) status to the clusters. Taxonomic classification is based on NCBI Taxonomy^20^ (http://www.ncbi.nlm.nih.gov/taxonomy). A total of 11795 sequences, where the biggest was 1768 bp and the smallest 1200 bp, was used for the reference database.

## RESULTS AND DISCUSSION

### Improved hydrogen gas mass transfer led to an increase in current production

**Figure 2:**
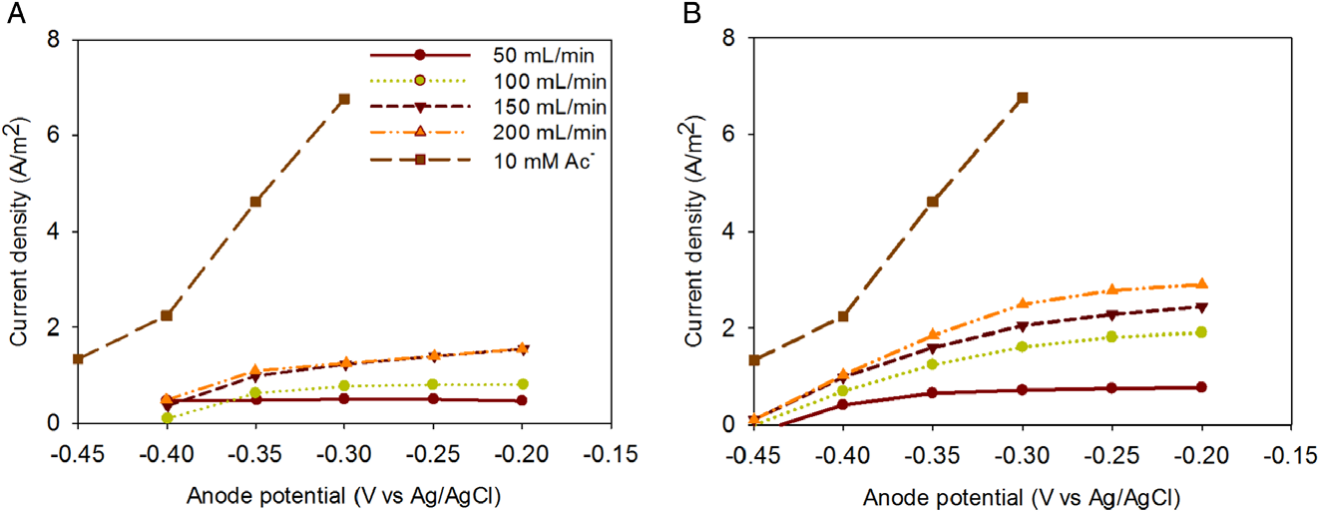
Current density production at an inflow of hydrogen gas of 3 mL min^‐**1**^ (A) and 10 mL min^‐**1**^ (B) for anolyte circulation speed of 50, 100,150 and 200 mL min^‐**1**^, compared with the current produced in the experiment with 10 mmol L^‐**1**^ acetate.

To assess the improvement in hydrogen gas oxidation at a bioanode by using a GDE, current densities at different anode potentials were evaluated. Figure 2 shows the current density produced with acetate and hydrogen gas as an electron donor at different hydrogen gas inflow rates and anolyte recirculation speed. For acetate, the maximum current density was 6.8 A m^**‐**2^ at an anode potential of –0.30 V, which is comparable with values reported in the literature.^3^, ^4^, ^21^ For hydrogen gas as electron donor supplied at 3 mL min^‐1^, the maximum current density was 1.5 A m^**‐**2^ at –0.20 V anode potential, for the highest anolyte recirculation speed of 200 mL min^‐1^. For hydrogen gas supplied at 10 mL min^‐1^, an increase in current density was observed, with a maximum current density of 2.9 A m^‐2^ at –0.20 V anode potential at 200 mL min^‐1^ anolyte recirculation. From the figures, three trends can be seen: first, an increase in hydrogen gas inflow rate led to higher current production due to better mass transfer from the gas phase to biofilm; second, the current density increased at higher overpotentials for both inflow rates; and third, an increase in the recirculation speed of the anolyte resulted in the higher current, which indicates that mass transfer limitations arise from dissolved species in the anolyte. The transport of protons away from the biofilm may be limiting the current density, as has been observed for acetate oxidation.^11^, ^12^, ^22^ Control experiments performed without biofilm on graphite felt with platinum as current collector revealed that no current was produced in the used potential range (data not shown).

Compared with the previous study that used hydrogen gas at the bioanode, the current improved roughly 2‐fold.^9^ Although the results are not comparable one to one because of different materials used in the two studies, this still gives an indication of the improvement for hydrogen oxidation at the set anode potentials.

The current produced from hydrogen gas was much lower when compared with acetate oxidation at bioanodes. This fact is not only reflected in the current density produced at different anode potentials but also becomes visible in the open circuit potentials. Theoretically, for hydrogen gas oxidation, the equilibrium potential is –0.62 V at pH 7, while for acetate the equilibrium potential is –0.50 V at pH 7. The measured open circuit potential for acetate, during start‐up and first characterization phase, was –0.48 V, which is close to the theoretical value. For hydrogen gas oxidation, however, the measured open circuit potential was around –0.45 V; a value much further from thermodynamic equilibrium. Thus, the overpotential for hydrogen gas oxidation is much higher than for acetate oxidation.

### Effect of bicarbonate addition on bioanode performance

As bioanode performance for hydrogen gas oxidation was considerably lower than bioanode performance for acetate oxidation, we investigated if the production of electricity from hydrogen could be improved by acetate as an intermediate component. Acetate can first be produced from hydrogen gas and bicarbonate and subsequently oxidized to electrons and bicarbonate. To study if acetate could be a suitable intermediate for electricity generation, we investigated bioanode performance at different bicarbonate concentrations (1 mmol L^**‐1**^ to 50 mmol L^**‐1**^) and 10 mL min^**‐1**^ H_2_ supply. These experiments were performed at an anode recirculation speed of 100 mL min^**‐1**^.

**Figure 3:**
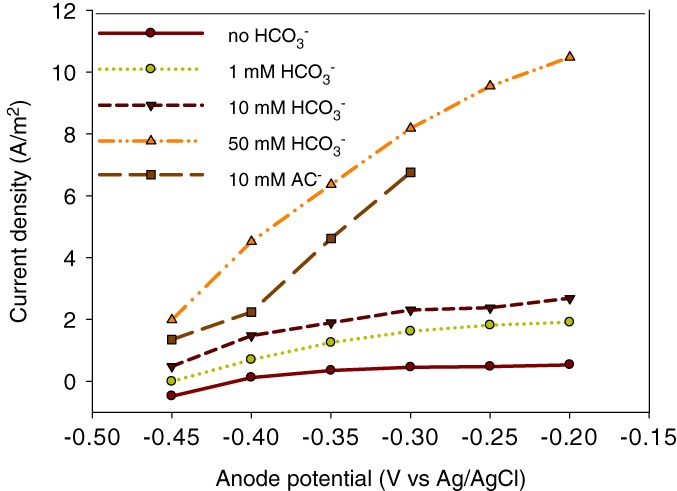
Current density produced by the bioanode fed with hydrogen gas (10 mLmin^‐**1**^) as a function of the controlled anode potential at different bicarbonate concentrations (0–50 mmol L^‐**1**^), compared with the experiment with acetate.

Figure 3 shows the effect of different bicarbonate concentrations on current density at different anode potentials. With increasing bicarbonate concentrations, we observed an increase in current density. The current densities increased to 8.2 A m^**‐**2^ for 50 mmol L^**‐1**^ bicarbonate and 6.9 A m^**‐**2^ for acetate at ‐–30 V. During these polarization tests, acetate was detected in the anolyte. After 1 day of operation, the acetate concentration was 0.049 mmol L^**‐1**^ for 1 mmol L^**‐1**^ bicarbonate, 0.13 mmol L^**‐1**^ for 10 mmol L^**‐1**^ bicarbonate, and 1.9 mmol L^**‐1**^ for 50 mmol L^**‐1**^ bicarbonate, showing that acetate was produced from H_2_ and bicarbonate.

The results presented in Fig. 2 were obtained when the cell was fed with 1 mmol L^**‐1**^ HCO_3_^‐^, which raises the question if acetate was also formed as intermediate in those experiments. Therefore, an additional experiment was performed where no HCO_3_^‐^ was fed to the bioanode. In this case, the maximum current density was 0.53 A m^**‐**2^ at an anode potential of –0.20 V (Fig. 3), showing that hydrogen gas oxidation can occur in the absence of bicarbonate (and acetate) which is in accordance with the findings in a previous study by Ntagia *et al*.^9^

### Improved mass transfer in anolyte led to higher current density

**Figure 4:**
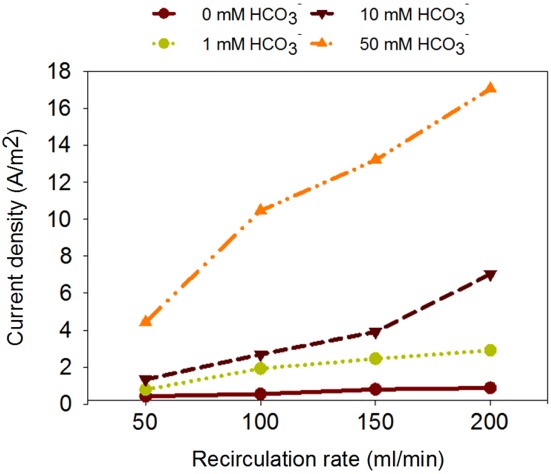
Current density at a controlled anode potential of –0.20 V vs Ag/AgCl potential at a hydrogen gas inflow rate of 10 mL min^‐**1**^ at different bicarbonate concentrations (0–50 mmol L^‐**1**^) as a function of the anolyte recirculation speed (50–200 mL min^‐**1**^).

To study the current produced from hydrogen gas at different bicarbonate levels, polarization tests were performed at different recirculation rates. Figure 4 shows the relation between recirculation speed, bicarbonate concentration and current density at –0.20 V anode potential. This figure shows an increase in current density with increasing recirculation rate for the three bicarbonate concentrations, and shows that enhanced mass transfer in solution improves the conversion rate. Figure 2 already showed a slight increase in current production at higher circulation speed and higher hydrogen flow rate. This increase, however, was only effective for a limited anode potential range (to –250 mV). Therefore, the increase in current density in Fig. 4 can only be partly explained by improved mass transfer of hydrogen gas to the bioanode. The further increase in current production is due to improved mass transfer of the acetate produced to the biofilm and protons produced away from the biofilm. At a recirculation rate of the anolyte of 200 mL min^**‐1**^, the current density achieved a maximum of 17 A m^**‐**2^ at –0.200 V and 50 mmol L^**‐1**^ bicarbonate.

### Mechanisms for current production from hydrogen gas and bicarbonate

The overall experiment was set up to improve the performance of a hydrogen oxidizing bioanode by improving the mass transfer using a gas diffusion electrode. The inoculum was taken from an acetate‐oxidizing bioanode from an active MFC, with *Geobacter* sp as the expected dominant electro‐active species. This inoculum was used because it is known that *Geobacter* sp can oxidize both acetate and H_2_.^9^ Interestingly, after increasing bicarbonate levels, acetate was produced and detected in our bioanode even though the bioanode was inoculated with an enriched culture of electrogens and not with specific acetogenic microorganisms. It has been suggested, though, that a specific inoculum is required for acetate production in biocathodes.^23^ According to our results, under certain conditions, with sufficiently high H_2_ pressure and a sufficiently high HCO_3_^‐^ concentration, acetate formation will take place even if the reactors was not inoculated with specific acetogens. Analysis of the microbial community at the end of the experiment revealed the presence of both *Geobacter* sp and Acetobacterium sp. (abundance of 34 and 23%, respectively). *Geobacter* is known to be able to convert both acetate and H_2_ into electrons, while Acetobacterium is known to produce acetate. Due to the presence of both acetate and hydrogen gas, it is difficult to determine the mechanisms through which the electrons are released to the anode. Four possible scenarios can be distinguished.

In the first scenario, bicarbonate serves as carbon source, while hydrogen gas serves as electron donor, while the acetate formed has no effect on the generated current. This scenario would require the presence of autotrophic electroactive cultures where bicarbonate is the carbon source for the biomass. Ntagia *et al*. showed that hydrogen gas was directly oxidized at a bioanode with no detectable organic compounds in the anolyte,^9^ a finding that was also observed in this study at 0 mmol L^**‐1**^ bicarbonate. However, the current density generated in Ntagia *et al*. could only be sustained for a limited period of time (two weeks) when no additional carbon source was added, showing that the activity of electroactive autotrophs was dependent on carbon availability. Therefore, the direct oxidation of hydrogen gas might have determined the generated current. However, the presence of acetate likely results in a different scenario.

In a second scenario, hydrogen gas is directly oxidized by the biofilm to generate current while the acetate produced functions as a carbon source for the hydrogen oxidizing bacteria, keeping the biomass population active on the electrode surface. Visser showed that in sulfate reducing reactors, at increasing organic COD concentrations, e.g. acetate, an increase of hydrogen oxidation could be observed.^24^ Translated to a bioanode, this would mean that the hydrogen oxidizers would show better performance in the presence of acetate, as growth rates are higher.

In the third scenario, hydrogen gas is first converted together with HCO_3_^‐^ into acetate by acetogens, after which acetate is oxidized at the bioanode by the electrogens to electricity.^25^, ^26^ Although direct oxidation of hydrogen at the anode is thermodynamically more favorable than the conversion of hydrogen gas to acetate, the acetogens have a spatial advantage compared with electrogens to use hydrogen, since they are not limited to the electrode and can live freely in solution.

In the fourth and final scenario, hydrogen gas and acetate are both used by the same microbial community to produce electricity. *Geobacter* can oxidize both substrates independently, while acetate can also be used as a carbon source for growth during hydrogen oxidation. This dual electron donor oxidation process has also been observed in BES when hydrogen and acetate are both present in solution.^27^, ^28^

## PERSPECTIVES

In this study it was shown that, at an anode potential of –0.3 V, hydrogen gas oxidation produced 1.6 A m^**‐**2^, while acetate oxidation alone produced 6.8 A m^**‐**2^. A combination of hydrogen oxidation and 50 mmol L^**‐1**^ bicarbonate addition resulted in 8.3 A m^**‐**2^. Increase of the anode potential to –0.2 V produced a current density of 2.9 A m^**‐**2^ while at a bicarbonate concentration of 50 mmol L^**‐1**^ and enhanced mixing of the anolyte 17.1 A m^**‐**2^ was produced.

The extent to which acetate can act as an intermediate for current production from hydrogen gas, of course, depends on the availability of bicarbonate. In our experiments, acetate formation was already observed at 1 mmol L^**‐1**^ HCO_3_^‐^, Bicarbonate is available at concentrations between 1 and 5 mmol L^**‐1**^ in groundwater.^29^ At mining locations, it is common to find water with high hardness (50 to 500 mmol L^**‐1**^) that degasses slowly due to the acidification or solubilized suspended carbonates when pH decreases.^30^ Regulations do not allow water hardness in concentrations >5 mmol L^**‐1**^ in surface or tap water. Therefore, mining water treatment should include processes to reduce carbonate and bicarbonate content.^31^, ^32^ Its use for acetate production, in combination with use of H_2_ that is otherwise burned, could present a solution. Hydrogen gas may thus offer opportunities as electron donor for bioanodes in situations where excess hydrogen and no organics are available. Kinetics of hydrogen gas oxidation, however, remain slow, even when a gas diffusion electrode is used. Production of acetate from H_2_ and HCO_3_^‐^ might be an interesting route to improve kinetics of the conversion of hydrogen gas into electricity.
